# Tim-3 Negatively Mediates Natural Killer Cell Function in LPS-Induced Endotoxic Shock

**DOI:** 10.1371/journal.pone.0110585

**Published:** 2014-10-22

**Authors:** Hongyan Hou, Weiyong Liu, Shiji Wu, Yanjun Lu, Jing Peng, Yaowu Zhu, Yanfang Lu, Feng Wang, Ziyong Sun

**Affiliations:** 1 Department of Clinical Laboratory, Tongji Hospital, Tongji Medical College, Huazhong University of Science and Technology, Wuhan, China; University of Colorado School of Medicine, United States of America

## Abstract

Sepsis is an exaggerated inflammatory condition response to different microorganisms with high mortality rates and extremely poor prognosis. Natural killer (NK) cells have been reported to be the major producers of IFN-γ and key players in promoting systematic inflammation in lipopolysaccharide (LPS)-induced endotoxic shock. T-cell immunoglobulin and mucin domain (Tim)-3 pathway has been demonstrated to play an important role in the process of sepsis, however, the effect of Tim-3 on NK cell function remains largely unknown. In this study, we observed a dynamic inverse correlation between Tim-3 expression and IFN-γ production in NK cells from LPS-induced septic mice. Blockade of the Tim-3 pathway could increase IFN-γ production and decrease apoptosis of NK cells in vitro, but had no effect on the expression of CD107a. Furthermore, NK cell cytotoxicity against K562 target cells was enhanced after blocking Tim-3 pathway. In conclusion, our results suggest that Tim-3 pathway plays an inhibitory role in NK cell function, which might be a potential target in modulating the excessive inflammatory response of LPS-induced endotoxic shock.

## Introduction

Sepsis is characterized by an exaggerated systemic inflammatory response mainly caused by lipopolysaccharide (LPS) of Gram-negative bacterium, leading to serious effects such as multi-organ failure and even death [1]. The overwhelming release of proinflammatory cytokines, in particular TNF-α and IFN-γ, are involved in the development of sepsis [2]. Thus, strategies aimed at down-regulating the excessive inflammatory condition may be potentially useful for therapy of sepsis. Previous studies have indicated that macrophages, neutrophils and conventional T cells are activated and contribute to the sepsis-induced systemic inflammatory response [3]. Natural killer (NK) cells, which have been identified as the major producers of IFN-γ, also play a central role in the pathogenesis of sepsis. Depletion of NK cells provides protection against LPS or multi-bacteria-induced sepsis in mice [4]–[6].

T-cell immunoglobulin and mucin domain (Tim-3), a type I membrane glycoprotein, has been reported to be expressed on activated CD4^+^ T cells, CD8^+^ T cells, monocytes, dendritic cells (DCs) and NK cells [7]–[10]. Engagement of Tim-3 with its ligand galectin-9 [11] has been reported to play important roles in various immune responses such as infection, autoimmunity, and tumor immunity [12]–[14]. Moreover, the high expression of Tim-3 mRNA was observed in human NK cells when compared with other lymphocyte populations [15]. Previous studies have shown that Tim-3 acts as an activating coreceptor of human NK cells to enhance IFN-γ production among healthy individuals [16], [17]. In contrast, Tim-3 pathway might have different influence on NK cell function in patients with hepatitis B virus infection and atherogenesis, in which upregulation of Tim-3 on NK cells correlates with decreased IFN-γ production and cytotoxicity [10], [18].

Tim-3 has also been proved to negatively regulate the toll-like receptor 4 (TLR-4)-mediated immune responses and plays important roles in maintaining the homeostasis of sepsis [19]. Our previous study also found that Tim-3 pathway could regulate LPS-induced endotoxic shock through CD4^+^ T cells, CD8^+^ T cells, and NK cells [20]. However, the precise mechanism by which the Tim-3 pathway regulates the phenotype and function of NK cells in sepsis still remains largely unknown. In this study, we dynamically detected the expression of Tim-3 on peritoneal NK cells during the development of LPS-induced endotoxic shock and further assessed its effect on NK cell activity. Our findings support the inhibitory role of Tim-3 on NK cells in LPS-induced endotoxic shock.

## Materials and Methods

### Mice

BALB/c mice (male, 6–8 weeks of age, weight 20–25 g) were purchased from Experimental Animal Center of Tongji Medical College, Huazhong University of Science and Technology, Wuhan, China. All mice were bred under specific pathogen-free conditions at Tongji Hospital animal facility. All experimental procedures on animals used in this study were carried out according to the protocol approved by the Institutional Animal Care and Use Committee at the Tongji Medical College. All surgery was performed under sodium pentobarbital anesthesia (50 mg/kg, i.p.), and all efforts were made to minimize animal discomfort.

### Reagents and Abs

Abs to CD3 (11–0031), NKp46 (11–3351; 47–3351), Tim-3 (12–5871), CD69 (15–0691), IFN-γ (17–7311; 11–7311), CD107a (50–1071), granzyme B (50–8898), perforin (17–9392), CD4 (11–0042), CD8 (11–0081), CD11b (11–0112), CD11c (11–0114), F4/80 (11–4801), and Annexin V-PI Apoptosis Detection Kit (88–8007) were purchased from eBioScience (San Diego, CA). Anti-galectin-9 (136103) was purchased from Biolegend (San Diego, CA). Anti-Tim-3 blocking antibody (anti-Tim-3 Ab) (clone 8B.2C12; 16–5871) was purchased from eBioScience. Recombinant mouse Tim-3 Fc protein (1529-TM-050) was purchased from R&D Systems (Minneapolis, MN). Anti-CD3 microbead kit (130-094-973) and anti-NKp46 microbead kit (130-095-390) were purchased from Miltenyi Biotec (Miltenyi Biotec, GmbH). LPS (E. coli O55:B5) was obtained from Sigma-Aldrich, dissolved in PBS and stored at4°C

### Induction of experimental sepsis

Specific pathogen-free BALB/c mice were used to establish the LPS-induced sepsis model. Mice were injected i.p. with 15 mg/kg of LPS, as with previously described methods [20].

### Cell preparation and culture

Mice were euthanized by cervical dislocation after sodium pentobarbital anesthesia (50 mg/kg, i.p.) at 24 h post-LPS injection. Spleen cells were separated through density gradient by mouse lymphocyte separation medium (DKW33-R0100). Spleen cells were plated at 1×10^5^ cells/well in 96-well plates in RPMI 1640 supplemented with 10% fetal calf serum (FCS) and stimulated with LPS (1 µg/ml) in the presence of anti-Tim-3 Ab (1 µg/ml), Tim-3 Fc protein (5 µg/ml) or control IgG. Cell suspensions were cultured in 5% CO_2_ incubator for 24 h, and then the cells were collected and analyzed by flow cytometry. For the analysis of intracellular IFN-γ production, monensin (1 µM, eBioScience) was added to cultures for the last 6 h of incubation.

### Flow cytometry

Mice were euthanized by cervical dislocation after sodium pentobarbital (50 mg/kg, i.p.) anesthesia every 4 h in the initial 24 h after LPS injection. Peritoneal cells were harvested by peritoneal lavage using 10 ml PBS and the total numbers of cells were counted. Erythrocytes were removed by cell lysis in FACS lysing solution (BD Biosciences, Heidelberg, Germany). Peritoneal cells and spleen cells were pre-incubated for 10 min at room temperature in 10% FCS to block Fc receptors and non-specific binding. After washing three times, cells were stained with Abs specific for mouse CD3, NKp46, Tim-3, CD69, CD107a, CD4, CD8, CD11b, CD11c, F4/80, galectin-9 and incubated on ice for 30 min. For intracellular staining, the cells collected after surface staining were fixed and permeabilized with Fixation and Permeabilization Buffer (BD Pharmingen). After permeabilization, cells were stained with Abs to IFN-γ, granzyme B, perforin and analyzed using a FACScan flow cytometer (Becton Dickinson). NK cell apoptosis was assessed using an Annexin V-PI Apoptosis Detection Kit according to the manufacturer’s instructions. Data analysis was performed using FlowJo version 7.6.1 software (TreeStar).

### NK cell cytotoxicity analysis

NK cells were purified from the spleen cells of septic mice at 24 h after LPS injection by MACS according to the instructions of the manufacturer (Miltenyi Biotec). Briefly, CD3^+^ cells were depleted from spleen cells with magnetic beads conjugated to anti-CD3, and from the negative cell fraction, NKp46^+^ cells were isolated by positive selection with magnetic beads conjugated to anti-NKp46. The purity of NK cells (CD3^−^NKp46^+^) was more than 95% as assessed by flow cytometry. Purified NK cells were incubated with anti-Tim-3 Ab (1 µg/ml) or control IgG for 18 h. After incubation, the NK cells were collected as effector cells. Then carboxyfluorescein succinimidyl ester (CFSE) labeled K562 target cells were added to the cultures at effector: target (E: T) ratios of 0∶1 (negative control) and 10∶1 (test) and incubated at 37°C for additional 6 h. Immediately before acquisition, PI was added to each tube and the cells were analyzed by flow cytometry.

### Statistical analysis

Data are expressed as the mean ± standard errors of measurement (SEM). Differences between groups were analyzed using two-tailed Student’s *t*-test. GraphPad Prism (version 5.01, GraphPad) software was used for all statistical procedures. Values of *p*<0.05 were considered as statistically significant.

## Results

### The dynamic expression of Tim-3 and intracellular IFN-γ in NK cells during the course of LPS-induced endotoxic shock

**Figure 1:**
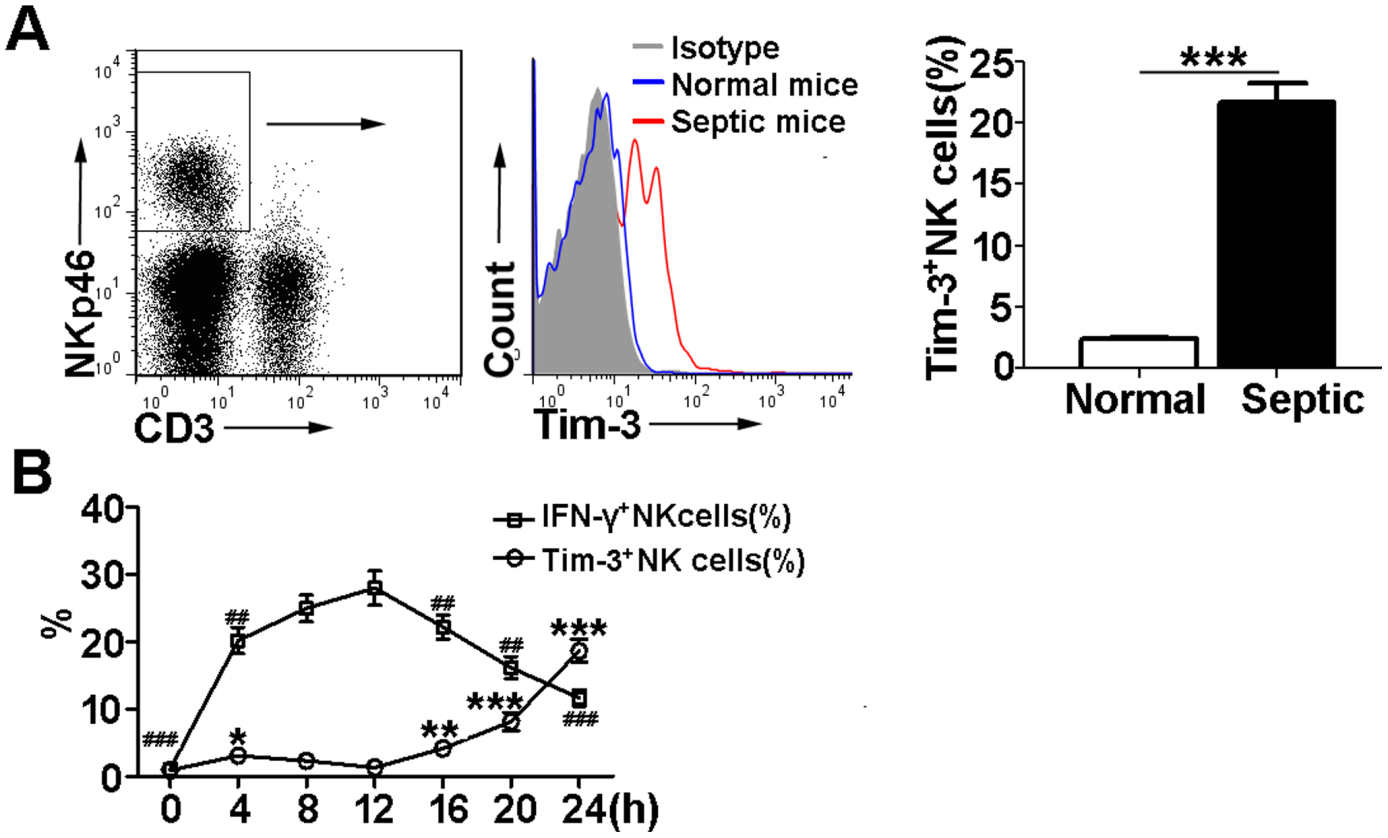
Expression of Tim-3 and IFN-γ in NK cells during the development of LPS-induced endotoxic shock. Peritoneal cavity cells were collected from normal and septic mice at different time points after LPS injection and were analyzed by flow cytometry. (A) NK cells were gated as the CD3^−^NKp46^+^ population. Representative flow cytometry histograms showed Tim-3 expression on NK cells from normal and septic mice at 24 h after LPS injection. The percentage of Tim-3^+^ NK cells was shown in the bar graphs. (B) The percentages of Tim-3^+^ NK cells and IFN-γ^+^ NK cells from septic mice at different time points after LPS injection were shown. Data are mean ± SEM of at least three independent experiments. *p<0.05, **p<0.01, ***p<0.001 compared with septic mice at 0 h after LPS injection; ^##^ *p*<0.01, ^###^ *p*<0.001 compared with septic mice at 12 h after LPS injection.

Tim-3 pathway has been described to play important roles in immune regulation of sepsis [19], [20]. Our previous data have suggested that Tim-3 signaling pathway serves as a novel negative mediator in the development of sepsis and that the expression of Tim-3 is increased on NK cells [20]. In this study, we further determined the effect of Tim-3 on NK cell function during the development of sepsis. The expression of Tim-3 and intracellular IFN-γ was examined in peritoneal NK cells at different time points after LPS injection. Our results showed that the expression of Tim-3 on NK cells was significantly increased at 24 h after LPS injection (Fig. 1A). Furthermore, we observed that peritoneal NK cells exhibited low levels of Tim-3 and IFN-γ expression in the initial inflammatory response to LPS infection. Tim-3 expression on NK cells had a moderate increase at 4 h but declined to undetectable levels at 12 h after LPS injection. Meanwhile, the expression of IFN-γ in NK cells was steadily elevated and reached its peak at 12 h. In the following 12–24 h, the expression of Tim-3 on NK cells was increased gradually, while the expression of IFN-γ in NK cells was decreased (Fig. 1B). The percentages and absolute numbers of Tim-3^+^ NK cells and IFN-γ^+^ NK cells in the peritoneal cavity of septic mice at different time points are shown in Table 1. These data highlight a dynamic inverse correlation between Tim-3 expression and IFN-γ production of NK cells in LPS-induced endotoxic shock.

**Table 1:** The percentages and absolute numbers of Tim-3^+^ NK and IFN-γ^+^ NK cells.

	Tim-3^+^ NK %	(numbers ×10^4^ cells)	IFN-γ^+^ NK %	(numbers ×10^4^ cells)
0 h	0.880±0.192	(2.16±0.109)	1.09±0.339^###^	(2.68±0.042^###^)
4 h	3.05±0.202^*^	(6.98±0.150^***^)	20.6±1.09^##^	(47.2±1.01^##^)
8 h	2.30±0.178	(5.13±0.017^***^)	24.4±1.51	(54.4±2.39)
12 h	1.30±0.435	(2.83±0.041^**^)	27.9±0.897	(60.8±2.63)
16 h	4.13±0.278^**^	(8.51±0.231^***^)	23.0±0.505^##^	(47.4±1.29^##^)
20 h	8.15±0.362^***^	(16.1±0.644^***^)	16.4±2.42^##^	(32.5±1.30^###^)
24 h	17.5±2.64^***^	(33.8±2.09^***^)	11.3±1.37^###^	(21.9±1.36^###^)

Data are mean ± SEM of at least three independent experiments. *p<0.05, **p<0.01, ***p<0.001 compared with septic mice at 0 h after LPS injection; ^##^ *p*<0.01, ^###^ *p*<0.001 compared with septic mice at 12 h after LPS injection.

### The relationship between Tim-3 expression and NK cell activity in LPS-induced endotoxic shock

**Figure 2:**
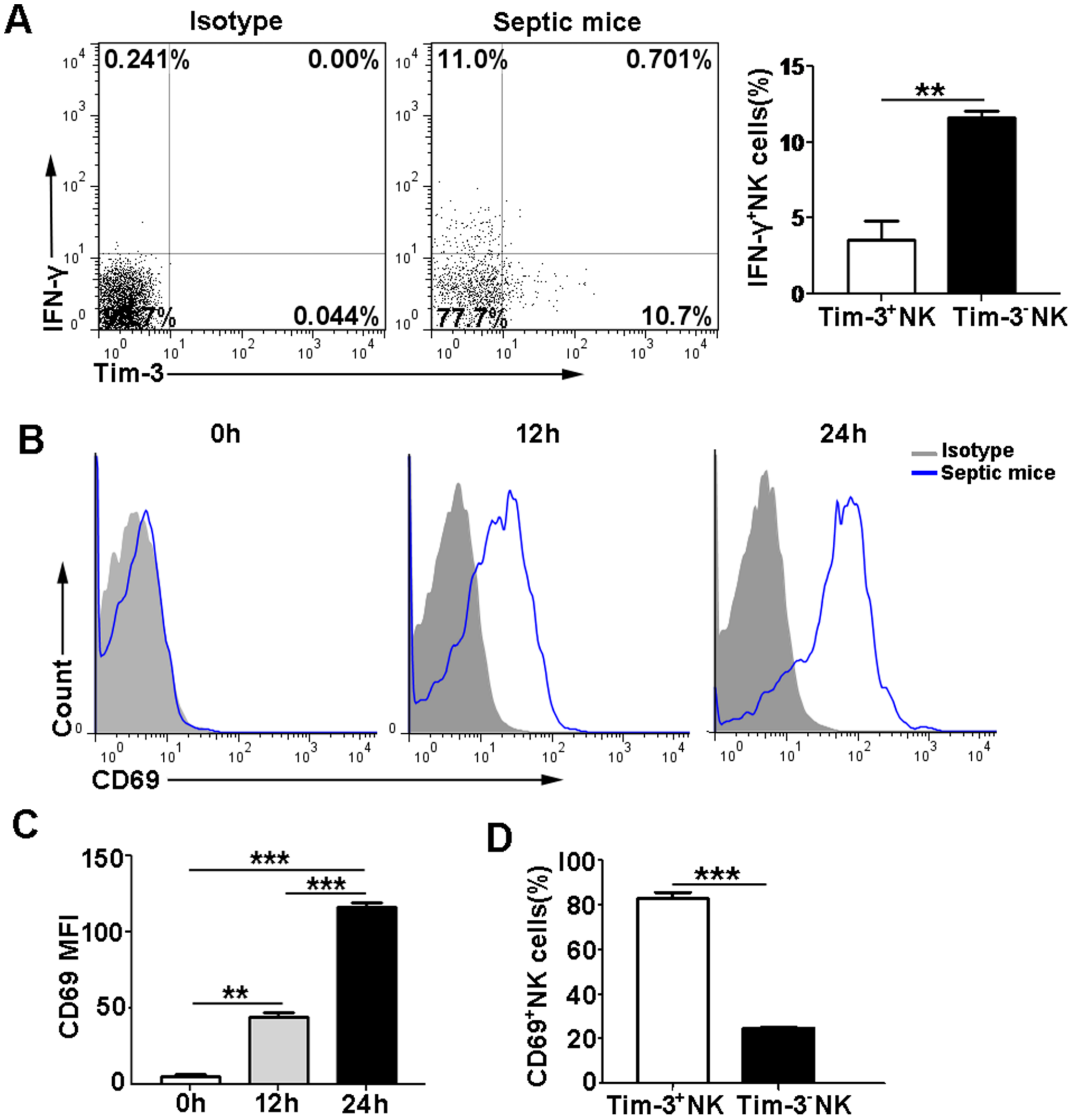
Tim-3 expression is inversely associated with NK cell activity. (A) The expression of Tim-3 and IFN-γ was analyzed in NK cells from septic mice at 24 h after LPS injection. The percentage of IFN-γ^+^ cells between Tim-3^+^ and Tim-3^−^ NK cell subsets was shown in the graphs. (B) Representative flow cytometry histograms of CD69 expression on NK cells from septic mice at 0, 12 and 24 h after LPS injection were shown. (C) The MFI of CD69 on NK cells was shown in the bar graphs. (D) The percentage of CD69^+^ NK cells between Tim-3^+^ and Tim-3^−^ NK cell subsets from septic mice at 24 h after LPS injection was shown. Data are mean ± SEM of at least three independent experiments. **p<0.01, ***p<0.001.

To assess the effect of Tim-3 on NK cell function, we detected the activation and cytolytic effector molecules of NK cells from LPS-induced septic mice. Our data showed that Tim-3^−^ NK cells were the predominant subset that produced IFN-γ. We observed that Tim-3^−^ NK cells produced a higher percentage of IFN-γ compared with Tim-3^+^ NK cells (11.6±0.463% versus 3.58±1.21%; *p*<0.01) (Fig. 2A). CD69, as one of the early activation markers, was expressed on peritoneal NK cells and continuously increased within 24 h after LPS injection (Fig. 2B). We observed that the mean fluorescence intensity (MFI) of CD69 expression on NK cells was significantly increased in septic mice when compared with normal mice (Fig. 2C). In addition, the percentage of CD69-expressing cells in Tim-3^+^ NK cell subset was significantly higher than that in Tim-3^−^ NK cell subset (Fig. 2D).

**Figure 3:**
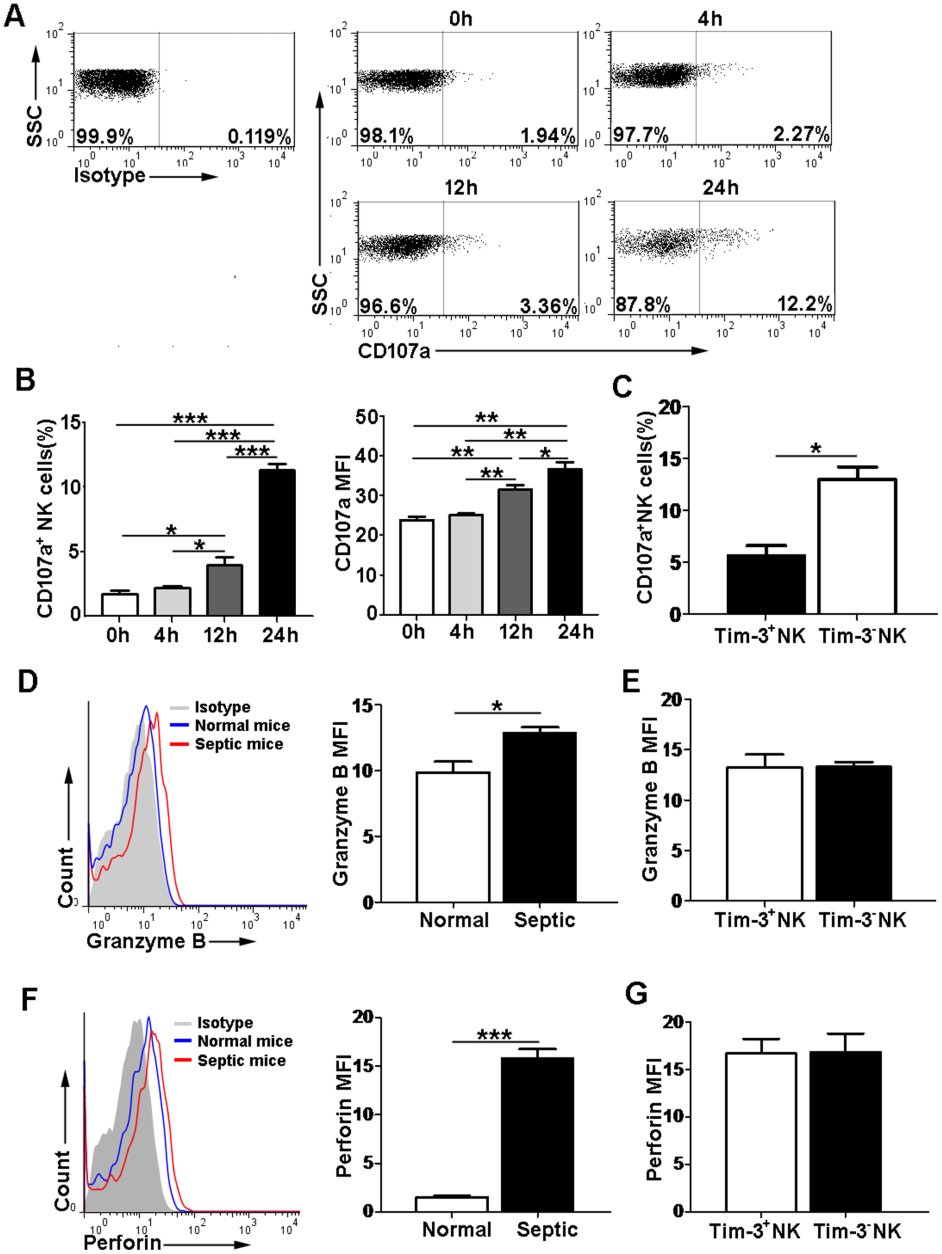
The relationship between Tim-3 expression and the cytotoxicity of NK cells. (A) Representative flow cytometric dot plots of CD107a expression on NK cells from septic mice at 0, 4, 12 and 24 h after LPS injection were shown. (B) The bar graphs showed the percentage and MFI of CD107a expression on NK cells at different time points. (C) The bar graphs showed the percentage of CD107a^+^ cells between Tim-3^+^ and Tim-3^−^ NK cell subsets from septic mice at 24 h after LPS injection. (D, F) Representative flow cytometry histograms showed granzyme B and perforin expression in NK cells from normal and septic mice at 24 h after LPS injection. The MFI of granzyme B and perforin expression in NK cells was shown. (E, G) The MFI of granzyme B and perforin expression in NK cells was compared between Tim-3^+^ and Tim-3^−^ NK cell subsets. Data are mean ± SEM of at least three independent experiments. *p<0.05, **p<0.01, ***p<0.001.

Furthermore, to evaluate whether the expression of Tim-3 correlated with the cytotoxic potential of NK cells, we directly examined the cell surface degranulation marker CD107a and intracellular cytotoxic effector molecules, including granzyme B and perforin. Our data showed that the expression of CD107a on NK cells was low at 0 h (1.70±0.282%) and 4 h (2.18±0.111%), but then increased at 12 h (3.99±0.547%) and reached up to approximately 11% (11.3±0.485%) at 24 h post-LPS injection (Fig. 3A). The percentage and MFI of CD107a expression on NK cells were significantly higher in septic mice than in normal mice (Fig. 3B). We also observed that Tim-3^−^ NK cells had higher expression of CD107a than Tim-3^+^ NK cells (Fig. 3C). In addition, we also detected the expression of cytotoxic effector molecules in NK cells and found that the MFI of granzyme B and perforin expression in NK cells was increased in septic mice at 24 h after LPS injection (Fig. 3D, F). We then compared the expression of these two cytotoxic effector molecules between Tim-3^+^ and Tim-3^−^ NK cell subsets, but they had no statistical significance (Fig. 3E, G). The above data indicated that Tim-3 pathway was negatively correlated with NK cell activity in LPS-induced endotoxic shock.

### Tim-3 blockade increases IFN-γ production and decreases apoptosis of NK cells

**Figure 4:**
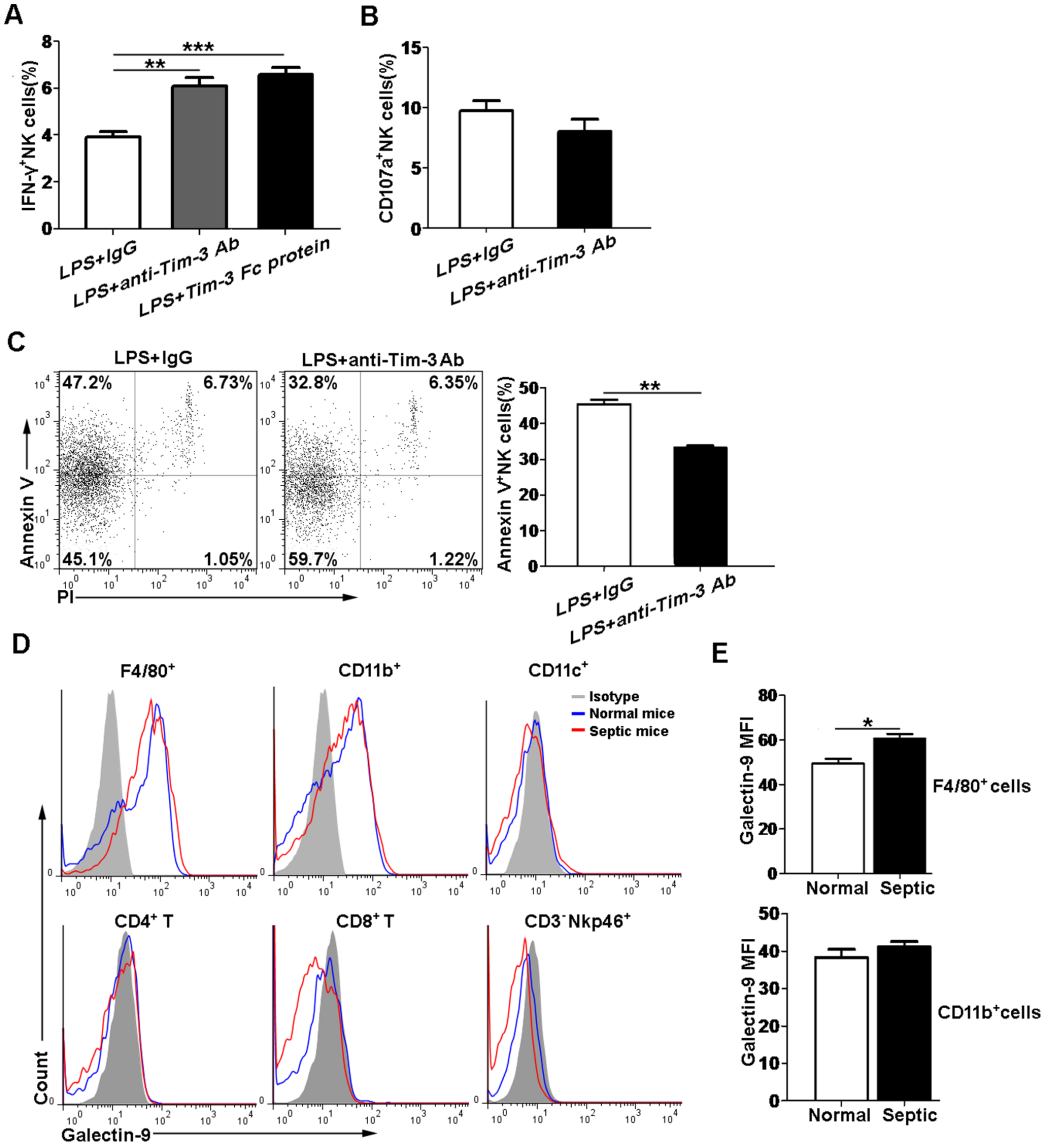
Blockade of Tim-3 pathway increased IFN-γ production and decreased apoptosis of NK cells in vitro. Spleen cells harvested from septic mice at 24 h after LPS injection were stimulated with LPS (1 µg/ml) in the presence of anti-Tim-3 Ab (1 µg/ml), Tim-3 Fc protein (5 µg/ml) or control IgG for 24 h. The bar graphs showed the percentages of (A) IFN-γ^+^ NK cells and (B) CD107a^+^ NK cells between the groups. (C) The apoptosis of NK cells (gated on CD3^–^NKp46^+^ cells) was analyzed by Annexin V/PI double staining. The percentage of Annexin V^+^PI^–^ cells (representative of apoptosis cells) was compared between the groups. (D) The expression of surface galectin-9 on peritoneal macrophages (F4/80^+^ cells), neutrophils (CD11b^+^ cells), DCs (CD11c^+^ cells), CD4^+^ T cells, CD8^+^ T cells and NK cells from normal and septic mice at 24 h after LPS injection were analyzed. (E) The MFI of galectin-9 on F4/80^+^ and CD11b^+^ cell populations was shown in the bar graphs. Data are mean ± SEM of at least three independent experiments. *p<0.05, **p<0.01, ***p<0.001.

To further confirm the relationship between Tim-3 expression and NK cell function, we made use of an anti-Tim-3 Ab to block Tim-3 signaling pathway in vitro. Our results showed that the percentage of IFN-γ^+^ NK cells had a near 2-fold increase after blocking Tim-3 pathway (Fig. 4A). On the other hand, we also used a recombinant Tim-3 Fc protein for interfering with Tim-3/Tim-3 ligand interaction. We observed that Tim-3 Fc protein had a similar effect with anti-Tim-3 Ab and could also significantly increase the production of IFN-γ by NK cells (Fig. 4A). However, no difference was observed in the expression of CD107a on NK cells after blockade of this pathway by using the anti-Tim-3 Ab (Fig. 4B). In addition, Annexin V/PI staining showed that blockade of Tim-3 pathway resulted in a significant decrease of NK cell apoptosis (Fig. 4C). These results suggested that blocking Tim-3 pathway could increase IFN-γ production and prevent the apoptosis of NK cells from LPS-induced septic mice.

Galectin-9, a ligand of Tim-3, has been reported to play important roles in regulating various inflammatory responses [12]–[14]. Thus, we also examined the surface galectin-9 expression on peritoneal lavage cells in this study. We observed that galectin-9 was expressed on both normal and septic mouse peritoneal macrophages and neutrophils, while it was not emerged on DCs, CD4^+^ T cells, CD8^+^ T cells and NK cells (Fig. 4D). The MFI of galectin-9 expression on macrophages was significantly increased in septic mice compared with that in normal mice. However, the expression of galectin-9 on neutrophils had no significant difference between the two groups (Fig. 4E). Our results demonstrated that Tim-3 ligand was present during sepsis, which suggested that Tim-3 signaling pathway might involve in the modulation of immune response of LPS-induced endotoxic shock.

### The cytotoxicity of NK cells is enhanced after blocking Tim-3 pathway

**Figure 5:**
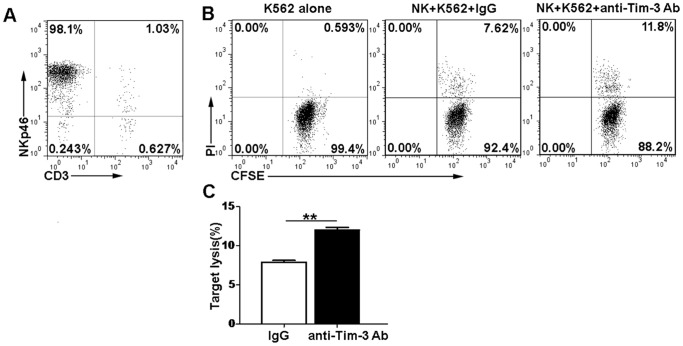
Blocking Tim-3 pathway enhances the cytotoxic activity of NK cells. NK cells were purified from the spleen cells of septic mice. Purified NK cells treated with anti-Tim-3 Ab (1 µg/ml) or control IgG for 18 h were used as effector cells. The effeteor cells were cocultured with CFSE-labeled K562 target cells at the E: T of 0∶1 and 10∶1 for 6 h. The death of target cells was detected by flow cytometry using PI staining. (A) The purity of NK cells was assessed by flow cytometric analysis of cells stained with anti-CD3 and anti-NKp46. (B) Representative flow cytometric dot plots showing the percentages of dead target cells in different experimental groups. (C) The percentage of target cell lysis was shown in the bar graphs. Data are expressed as the mean ± SEM of at least three independent experiments. **p<0.01.

We further determined the cytotoxic activity of NK cells after blockade of Tim-3 pathway. The cytotoxic activity of NK cells was tested for their ability to lyse the MHC class I-deficient K562 target cells. NK cells were isolated from spleen cells of septic mice and the purity of NK cells was more than 95% (Fig. 5A). Purified NK cells treated with anti-Tim-3 Ab or IgG control were used to lyse CFSE-labeled K562 cells. We observed that anti-Tim-3 Ab could significantly increase the cytotoxic activity of NK cells compared with IgG control (12.0±0.423% versus 7.77±0.327%; *p*<0.01) (Fig. 5B, C). These results indicated that Tim-3 might play an inhibitory role in NK cell-mediated cytotoxicity in LPS-induced endotoxic shock.

## Discussion

NK cells are major effector cells of the innate immunity and participate in the defense against microbial infections, involving IFN-γ secretion, target cell elimination and shaping the adaptive immune response [21], [22]. However, the function of NK cells are controversial in the process of sepsis [23]. It has been reported that depletion of NK cells can lead to significantly reduced inflammatory cytokine levels and improved survival of septic mice [4], [23]. Moreover, the increased number of NK cells in the peripheral blood of patients with severe sepsis was associated with an increased risk of mortality [24]. On the contrary, other researchers observed that high levels of NK cells were beneficial for the survival of septic patients [25], [26]. The discrepancies concerning the number and function of NK cells in septic patients are probably due to the heterogeneity of patients in terms of either severity or involvement of pathogens [25]. Thus, the mechanism of how NK cells regulate the immune response of sepsis still needs further investigation.

Sepsis is a life-threatening condition and a major cause of death in intensive care units, which is characterized by an overzealous release of proinflammatory cytokines and inflammatory mediators [27]. It suggests that appropriate immune status would be beneficial to counter the severe infectious processes. Previous studies have shown that the Tim-3 pathway plays important roles in regulating the inflammatory responses, such as autoimmune diseases [13], [28], transplant tolerance [29], [30], antitumor immunity [31], [32] and virus infection [12], [33], [34]. Tim-3 pathway has also been demonstrated to negatively regulate TLR-4 responses, resulting in down-regulation of the excessive inflammatory response and promoting the homeostasis of sepsis [19]. Our previous study have found that blockade of Tim-3 pathway can accelerate the death of septic mice [20], which suggests that Tim-3 serves as an important negative mediator in the development of LPS-induced endotoxic shock. Although our previous study mentioned the relationship between the upregulation of Tim-3 expression and the impairment of IFN-γ production in NK cells, the mechanism of Tim-3-mediated regulation of NK cell immunity during LPS-induced endotoxic shock still remains largely unknown. In the present study, we detected the dynamic expression of Tim-3 on NK cells and further determined the role of Tim-3 pathway in the regulation of NK cell function in LPS-induced endotoxic shock.

NK cells are the major producers of IFN-γ which can play an important role in regulating the process of experimental septic shock [4], [6]. Developmental studies show that mice deficient in IFN-γ are resistant to LPS-induced toxicity [35], and blockade of IFN-γ can improve the survival of septic mice [36]. In this study, we dynamically observed that the expression of Tim-3 on NK cells was inversely associated with IFN-γ production during the initial 24 h of LPS-induced endotoxic shock, which suggested that Tim-3 might act as an exhausted marker of NK cells in the process of the inflammatory response. In addition, both the use of anti-Tim-3 Ab and Tim-3 Fc protein could increase the secretion of IFN-γ by NK cells in vitro. To further determine the effect of Tim-3 pathway on sepsis, we also examined the expression of galectin-9, a known ligand for Tim-3 [11], and we observed that galectin-9 was indeed expressed on the surface of mouse peritoneal macrophages and neutrophiles. These data suggest that Tim-3 signaling pathway is involved in the down-regulation of NK cell immune response in LPS-induced endotoxic shock.

Previous studies regarding the function of NK cells in the pathogenesis of sepsis have focused on the production of cytokines rather than the cytotoxic capability. NK cells possess large amounts of cytolytic granules containing perforin and various granzymes and have the ability to kill the target cells directly. It has been reported that septic patients with increased levels of granzyme A and/or B have a higher mortality rate and more severe organ dysfunction [37]. Other researchers also observed that the elevated expression of granzyme B and perforin in the cytotoxic cells of septic patients correlated with disease severity [38]. As the data obtained regarding the evaluation of NK cell function are limited, we further assessed the cytotoxic activity of NK cells in LPS-induced endotoxic shock. CD107a, which is identified as a sensitive marker for degranulation of NK cells and activated CD8^+^ T cells, is significantly correlated with the cytotoxic activity of NK cells [39]. We observed that the expression of CD107a on NK cells was elevated in septic mice and that Tim-3^−^ NK cells had higher CD107a expression compared with Tim-3^+^ NK cells. These data suggested that Tim-3 was an exhausted marker of NK cells in LPS-induced endotoxic shock, which was also consistent with previous findings [40]. Furthermore, the MFI of granzyme B and perforin expression in NK cells was elevated in septic mice. This trend was consistent with the change of CD107a expression. However, there was no difference regarding the MFI of these two cytotoxic effector molecules between Tim-3^+^ and Tim-3^−^ NK cell subsets. The expression of CD107a on NK cells also had no statistical significance after blocking Tim-3 pathway. These data indicated that blockade of Tim-3 pathway could enhance IFN-γ production in NK cells but have little effect on NK cell degranulation in LPS-induced endotoxic shock. Nevertheless, the cytotoxic activity of NK cells against K562 cells was enhanced after blocking Tim-3 pathway. However, the precise mechanism by which Tim-3 blockade increased NK cell cytotoxicity needs to be further investigated.

In addition, extensive lymphocyte apoptosis has been reported in patients with sepsis [41], but the consequence of cell apoptosis in sepsis still remains ambiguous. Previous study has shown that lymphocyte apoptosis leads to immunosuppression and is associated with the mortality of septic patients [35]. On the other hand, other researchers have found that the reduced cell apoptosis of NK cells results in a further release of inflammatory cytokines and is harmful for the survival of septic shock patients [6]. Our previous results have shown that Tim-3^+^ T cells are more prone to apoptosis than Tim-3^−^ T cells in septic mice [20]. However, the effect of Tim-3 on the apoptosis of NK cells is still unknown. As expected, we observed that blockade of Tim-3 pathway could also reduce the apoptosis of NK cells from septic mice. These findings suggested that the increased expression of Tim-3 on NK cells might be associated with the down-regulation of inflammatory response, which could also be used to explain the mechanism of NK cell apoptosis in LPS-induced endotoxic shock.

Whereas Tim-3 expressed on normal human NK cells is proved to enhance IFN-γ production [16], [17], other studies suggest that Tim-3 expressed on NK cells may serve an opposite role in patients with HBV infection and atherosclerosis [10], [18]. Consistent with the latter observation, this study indicates that Tim-3 pathway plays an inhibitory role in NK cell function in LPS-induced endotoxic shock. This negative regulatory pathway represents a protective mechanism that can be used as a potential target in modulating the excessive inflammatory response of sepsis.
